# Differential Epigenetic Regulation of TOX Subfamily High Mobility Group Box Genes in Lung and Breast Cancers

**DOI:** 10.1371/journal.pone.0034850

**Published:** 2012-04-04

**Authors:** Mathewos Tessema, Christin M. Yingling, Marcie J. Grimes, Cynthia L. Thomas, Yushi Liu, Shuguang Leng, Nancy Joste, Steven A. Belinsky

**Affiliations:** 1 Lung Cancer Program, Lovelace Respiratory Research Institute, Albuquerque, New Mexico, United States of America; 2 Department of Internal Medicine, University of New Mexico, Albuquerque, New Mexico, United States of America; The Chinese University of Hong Kong, Hong Kong

## Abstract

Aberrant cytosine methylation affects regulation of hundreds of genes during cancer development. In this study, a novel aberrantly hypermethylated CpG island in cancer was discovered within the *TOX2* promoter. *TOX2* was unmethylated in normal cells but 28% lung (n = 190) and 23% breast (n = 80) tumors were methylated. Expression of two novel *TOX2* transcripts identified was significantly reduced in primary lung tumors than distant normal lung (p<0.05). These transcripts were silenced in methylated lung and breast cancer cells and 5-Aza-2-deoxycytidine treatment re-expressed both. Extension of these assays to *TOX*, *TOX3*, and *TOX4* genes that share similar genomic structure and protein homology with *TOX2* revealed distinct methylation profiles by smoking status, histology, and cancer type. *TOX* was almost exclusively methylated in breast (43%) than lung (5%) cancer, whereas *TOX3* was frequently methylated in lung (58%) than breast (30%) tumors. *TOX4* was unmethylated in all samples and showed the highest expression in normal lung. Compared to *TOX4*, expression of *TOX*, *TOX2* and *TOX3* in normal lung was 25, 44, and 88% lower, respectively, supporting the premise that reduced promoter activity confers increased susceptibility to methylation during lung carcinogenesis. Genome-wide assays revealed that siRNA-mediated *TOX2* knockdown modulated multiple pathways while *TOX3* inactivation targeted neuronal development and function. Although these knockdowns did not result in further phenotypic changes of lung cancer cells *in vitro*, the impact on tissue remodeling, inflammatory response, and cell differentiation pathways suggest a potential role for TOX2 in modulating tumor microenvironment.

## Introduction

Epigenetic inactivation of tumor suppressor genes is now established as one of the major mechanisms leading to the development and progression of cancer. Gene silencing through aberrant promoter CpG island hypermethylation is the most frequent epigenetic abnormality observed in various malignancies. To date, a number of genome-wide screening methods have been successfully employed to identify novel aberrantly methylated genes in cancer. These include: restriction landmark genomic scanning [1], CpG microarrays [2], [3], methyl-CpG binding domain chromatography [4], [5] and methylated CpG island amplification coupled with representational difference analysis (MCA/RDA) [6]. The MCA/RDA approach has been used to identify several methylated genes involved in colorectal [6], [7], [8], pancreatic [9], prostate, and breast cancers [10]. Previously, we used this assay to identify aberrant promoter CpG island methylation of the PAX5 alpha and beta transcription factors in human breast and lung cancers [11]. In this study the MCA/RDA was used to uncover a novel aberrantly methylated CpG island located in the promoter region of *TOX2*, a gene encoding for a high mobility group (HMG)-box protein.

HMG proteins are one of the most abundant chromatin-binding proteins that were initially characterized by high electrophoretic mobility in polyacrylamide gel. The HMG-box proteins are one of three classes of HMG proteins and are characterized by one or more HMG-box (a 70–80 amino acid DNA binding domain). Genetic and biochemical evidences indicate that the HMG-boxes of these proteins form three α-helices in a characteristic L-shaped structure that interacts with the minor grove of the DNA helix to promote bending and unwinding of compact chromatin [12], [13], [14]. Binding of HMG-boxes at the minor grove also allows simultaneous binding of transcription factors and other regulators required for DNA-based activities such as replication, transcription and DNA repair [12], [15]. The HMG-box family proteins are often divided into two subgroups based on their abundance and DNA binding specificity. The first group recognizes structural features of DNA with little or no sequence specificity, shows broad tissue distribution, and typically contains two or more HMG-box motifs (e.g. HMGB1–4). The second group binds DNA in a sequence specific manner, shows a more restricted expression pattern, contains one HMG-box domain, and consists of diverse proteins including TOX and SOX family members [16], [17], [18].

The recently introduced TOX subfamily consists of four genes, *TOX* (*TOX1*), *TOX2* (*GCX-1*, *C20orf100*), *TOX3* (*TNRC9*, *CAGF9*), and *TOX4* (*MIG7*) that share similar genomic structure and protein homology [16]. Although the HMG-box domains of these proteins show over 92% amino acid homology, the regions outside this domain are less conserved indicating non-overlapping functions. The limited functional assays available for these genes also support this supposition. TOX (for thymocyte selection-associated HMG-box) is primarily expressed in the thymus and regulates the differentiation programs of developing T-cells [19], [20]. Although the function of TOX2 in humans is not yet characterized, a rat ortholog of this gene with 100% HMG-box domain homology (GCX-1) is primarily expressed and functions in the hypothalamo-pitutary-gonadal axis of reproduction [21]. TOX3 is a neuronal survival factor that is highly expressed and regulates calcium dependent transcription in neurons [22], [23]. The expression profile and specific function of TOX4 is yet known, but this protein has been demonstrated to recognize DNA adducts specifically generated by platinum based anticancer drugs, suggesting it might function in DNA damage response and DNA repair pathways [24]. However, in contrast to a growing number of studies demonstrating abnormalities including aberrant promoter CpG island hypermethylation of multiple HMG proteins in various human malignancies, the role of TOX subfamily in carcinogenesis is unclear [25], [26], [27], [28], [29], [30], [31].

The purpose of this study was to perform a genome-wide comparison of DNA methylation between normal and tumor cells to identify novel methylation changes in cancer. Further studies focused on characterizing *TOX2*, a gene whose promoter CpG island was found to be specifically methylated in lung and breast cancer. The studies were also extended to other members of the *TOX* subfamily that share identical gnomonic structures with *TOX2* including a similarly located CpG island. The prevalence for aberrant methylation of these genes in primary lung and breast tumors, specificity of methylation to cancer cells, the effects of methylation on gene expression, and its reversibility with demethylating and chromatin regulating drugs were evaluated. The impact of epigenetic silencing of these genes on cancer properties such as cell proliferation, cell death, and cell migration were investigated. Finally, the genome-wide impact of epigenetic inactivation of *TOX* subfamily genes was evaluated using specific siRNAs to knock down individual genes, and genome-wide transcriptome arrays were used to define the genes and pathways affected by epigenetic silencing of this class of HMG-box proteins.

## Materials and Methods

### Tissue samples and cell lines

A total of 190 primary lung tumors were obtained from frozen tumor banks at Johns Hopkins, the Mayo Clinic, and St. Mary's Hospital (Grand Junction, CO). Distant normal lung tissues (DNLT) obtained from resected lung lobes of a subset of these samples were used as normal controls. Breast tumors and adjacent tissue were collected from women enrolled in a New Mexico Women's Health Study at the University of New Mexico. Non-malignant human bronchial epithelial cells (NHBEC) and peripheral blood mononuclear cells (PBMC) were obtained from cancer-free smokers at the New Mexico Veteran Health Care System. NHBEC were collected through diagnostic bronchoscopy and expanded in short-term tissue culture as described [32]. All samples were obtained with written informed consent from patients, and ethical approval of the study was granted by the Ethics Committee of the Lovelace Respiratory Research Institute. Five normal human bronchial epithelial cell lines (HBEC1, 2, 3, 13, and 14) immortalized as described [33] were obtained from Drs. Shay and Minna, Southwestern Medical Center, Dallas, TX. Twenty lung cancer cell lines (H23, H1435, H1568, H1993, H2023, H2085, H2228, H2009, H358, Calu-3, Calu-6, SKLU1, H1299, H1838, H1975, HCC827, HCC4006, A549, SW900, and H441), and four breast cancer cell lines (MCF-7, T47D, MDA-MB-231, and MDA-MB-435) were obtained from and authenticated by the American Type Culture Collection. Experiments were conducted in cell lines passed for a maximum of 6 months post-resuscitation.

### MCA/RDA

The MCA/RDA assay was performed exactly as described [11] using DNA from breast cancer cell lines (MCF-7, MDA-MB-231 and MDA-MB-435) as tester and DNA from normal breast tissue as driver. PCR products were ligated into the PCR II vector using the TA cloning kit (Invitrogen, San Diego, CA) and plasmid DNA containing the RDA products were prepared using the QIAprep Spin Miniprep kit according to the manufacturer's instructions (Qiagen, Valencia, CA). DNA sequencing was performed using a Cycle Sequencing Kit (USB) and samples were analyzed on a LICOR 4200 DNA Analyzer. Sequence homology was determined using the BLAST program of the National Center for Biotechnology Information (www.ncbi.nih.gov/BLAST).

### DNA methylation analysis

DNA extraction and modification were done exactly as described [34] and 40 ng of modified DNA was used per PCR. Methylation was first screened in NHBEC, PBMC, lung and breast cancer cell lines using Combined Bisulfite Modification and Restriction Analysis (COBRA) as described [34]. Methylation-specific PCR (MSP), developed and optimized using cell lines with defined methylation for each gene, was used to evaluate the methylation status of all samples including primary lung and breast tumors. Positive and negative control samples were included in each MSP assay. For selected samples the density and distribution of methylation across the CpG islands was assessed using bisulfite sequencing. Primer sequences and amplification conditions used for MSP, COBRA and sequencing assays are described in supporting information Table S1.

### Rapid amplification of cDNA ends (RACE)

RACE products (5′ and 3′) were produced using the GeneRacer RACE Ready Lung cDNA Kit (Invitrogen) using a 2-stage nested approach as recommended. The primer sequences and PCR amplifications conditions used for 5′ and 3′ RACE are shown in supporting information Table S2. First stage 5′ RACE products were generated using the gene specific primer GSP1 and the 5′ Gene Racer anchor primer GeneRacer™ 5′1 primer. Second stage 5′ RACE products were generated using the gene specific primer GSP2 and the 5′ Gene Racer nested anchor primer GeneRacer™ 5′ Nested primer. Similarly, first stage 3′ RACE products were generated using the gene specific primer GSP3 and the 3′ Gene Racer anchor primer GeneRacer™ 3′ primer. Second stage 3′ RACE products were generated using the gene specific primer GSP4 and the 3′ Gene Racer nested anchor primer GeneRacer™ 3′ Nested primer. All RACE products were analyzed on a 3% agarose gel containing ethidium bromide, visualized under UV illumination, cloned and sequenced.

### 5-Aza-2′-deoxycytidine (DAC) and trichostatin A (TSA) treatment

Lung cancer cell lines were maintained in ATCC-recommended media and cells at log-phase of growth were treated in duplicate as described [35] using Vehicle (0.6 µl ethanol in 10 ml medium), TSA (300 nM for 18 h [Sigma; stock solution 5 mM in ethanol]), or DAC (500 nM for 96 h with fresh medium containing the drug changed every 12 h [Sigma; stock solution 10 mM in PBS]). Cells treated with Vehicle or TSA underwent fresh media changes in parallel with DAC treatment. TSA treatment was conducted 18 h before all groups were harvested in TRI-Reagent (Sigma).

### Gene expression analysis

RNA was isolated as described [34] and 3 µg total RNA was reverse transcribed using the High Capacity cDNA Reverse Transcription Kit from Applied Biosystems (Foster City, CA) according to the manufacturer's protocol. To avoid PCR products from contaminating DNA, RNA isolation was done in the presence of DNase, and large introns were included in the RT-PCR amplification product. The effect of sham (Vehicle), TSA, and DAC treatments on gene expression was assessed using a gel-based assay as described [35]. RT-PCR primers and amplification conditions are described in supporting Table S1. TaqMan assays from Applied Biosystems, *TOX* (Hs00207075_ml), *TOX2* (Hs01031990_ml and Hs01040060_ml), *TOX3* (Hs01101330_ml), *TOX4* (Hs00927393_ml), and the housekeeping gene *beta-actin* (4310881E), were used for quantitative gene expression analysis. Each target gene was run at least twice in duplicate and the ΔCT values were generated from the housekeeping gene multiplexed in each reaction as the endogenous control. The ΔΔCT values were generated by comparing the reference samples to the test group, that is DNLT vs. primary tumors, and vehicle treated cell lines (control siRNA or vehicle) vs. cell lines treated with gene-specific siRNA or drugs (TSA or DAC) depending on the experiment. The relative gene expression levels were then calculated using the ΔΔCT method as described [36].

### Gene knock down and genome-wide expression analysis

Cell lines that are confirmed to express the gene of interest, *MDA-MB-231* (*TOX*), *Calu-3* and *MDA-MB-231* (*TOX2*), and *Calu-3* and *MCF-7* (*TOX3*) were transfected with negative control #1 (*siControl*) or gene-specific siRNAs, *TOX* s18842 (siTOX), *TOX2* s39780 (*siTOX2*), or *TOX3* s26152 (*siTOX3*) all from Applied Biosystems using Lipofectamine 2000 (Invitrogen, Santa Clara, CA). The impact of epigenetic down regulation of these genes on cell properties that include proliferation, cell death, and migration were compared between cells transfected with gene-specific or control siRNA using MTT and wound closure assays as described [37]. For genome-wide expression assays, cells were harvested 48 h post-transfection, gene knockdown was confirmed by TaqMan, and changes in gene expression was compared between *siControl* vs. *siTOX2* and *siControl* vs. *siTOX3* cells using the Agilent whole genome transcriptome array as described [35].

### Data analysis

Gene methylation and patient characteristics including age, gender, smoking status, tumor histology, and performance were summarized with mean and standard deviation for continuous variables and proportions for categorical variables. Survival time was calculated from time of diagnosis until death from any cause or last follow-up. The association between methylation and patient characteristics was assessed by Fisher's exact test. Kaplan-Meier plots, the log-rank test, and proportional hazards models were also employed. The effect of siRNA knockdown (*siControl* vs. *siTarget gene*) on gene expression was compared using one way analysis of variance (ANOVA). Tukey's and Dunnett's method were used for pair wise and treatment control comparison adjustments, respectively. The impact of potential outliers on the one way ANOVA values was controlled using nonparametric Wilcoxon Rank-sum test.

## Results

### MCA/RDA identifies a novel aberrantly methylated CpG island in cancer

Previously, we used the MCA/RDA technique developed by Toyota *et al* [6] to discover aberrant promoter hypermethylation of two transcription factor genes, *PAX5 α* and *β*, in human tumors [11]. Two other clones simultaneously discovered with the *PAX5* clone were homologous to the GenBank accession number AL035089, and map to chromosome 20q12-13.2 adjacent to each other at nucleotides 161, 665–161,987 and 161,982–162,220. They represent two consecutive 323 and 239 bp DNA segments that are flanked by three CCCGGG sequences, recognition sites for *SmaI* and *XmaI* restriction enzymes used in the MCA/RDA assay. These sequences were found to be hypermethylated in the breast cancer cell line (*MDA-MB-231*), but not in normal breast tissue. GenBank report for accession number AL035089 indicates the presence of a CpG island extending from nucleotide 160,344 to 162,383. This CpG island is GC-rich (0.71) with a CpG∶GpC ratio of 0.9, contains 216 CpGs, and is located in a typical promoter CpG island location spanning −394 to +1646 bp from the transcription start site of a gene encoding for a TOX high mobility group box protein, *TOX2*.

### *TOX2* promoter is hypermethylated in lung and breast tumors

**Figure 1:**
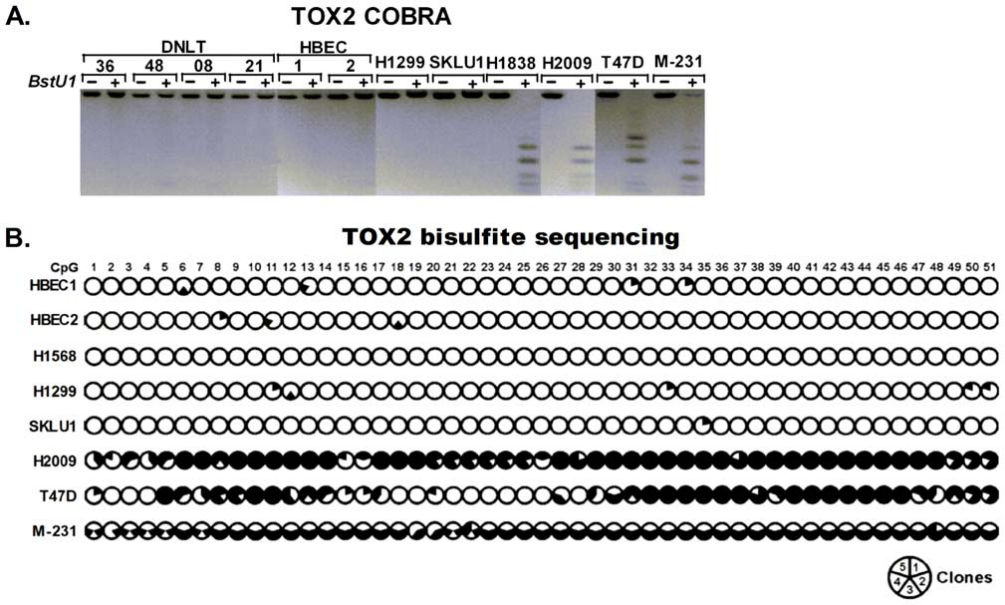
Methylation of *TOX2* promoter CpG island. **A**) Combined bisulfite modification and restriction analysis (COBRA) depicts methylation of *TOX2* promoter CpG island in normal and cancer samples. Complete, partial, or no methylation could be seen from digestion of all, some, or none of the PCR products in the presence of the *BstU1* (+) enzyme compared to no enzyme (−) control. MDA-MB-231 and MDA-MB-435 in all the figures are abbreviated as M-231 and M-435, respectively. **B**) Bisulfite sequencing was used to validate methylation results obtained through COBRA and MSP assays and to determine the degree and distribution of methylation at 51 CpG sites across *TOX2* promoter CpG island. Five clones were sequenced per sample and methylation status of each clone (1/5^th^ of a circle) at the specified CpG site is shown as methylated (filled) or unmethylated (open).

The presence and degree of methylation within *TOX2* promoter CpG island was first screened in lung and breast cancer cell lines using COBRA. Methylation was found in 4/20 (20%) lung cancer cell lines and 3/4 (75%) breast cancer cell lines (Table 1). In contrast, primary human bronchial epithelial cells (NHBEC) obtained from bronchoscopy of cancer free smokers (n = 20), five human bronchial epithelial cell lines (HBEC) immortalized as described [33], peripheral blood mononuclear cells (PBMC) obtained from cancer free donors (n = 10), and distant normal lung tissue (DNLT) obtained from NSCLC patients (n = 8) were unmethylated (Table 1 and Figure 1A). For selected samples the degree and distribution of methylation across the *TOX2* promoter CpG island was determined through bisulfite sequencing. The sequencing data validated results obtained through COBRA and MSP (not shown) assays and also revealed that the distribution of methylation across the 51 CpGs analyzed was mostly uniform (Figure 1B). Among primary tumors, *TOX2* methylation was detected in 28% (54/190) lung and 23% (18/80) breast tumors (Table 1). The prevalence for methylation of *TOX2* in lung cancer was similar between adenocarcinoma and squamous cell carcinoma. Interestingly, *TOX2* methylation among lung adenocarcinoma patients was significantly more prevalent in tumors from current smokers 43% (16/37) compared to never smokers 24% (18/75) or current non-smokers (former and never smokers combined) 26% (35/134) (p<0.05). Although not statistically significant, *TOX2* methylation in lung adenocarcinoma from current smokers was also higher than former smokers (43% vs. 29%, p = 0.15) (Table 1).

**Table 1:** Prevalence for promoter CpG island hypermethylation of *TOX* subfamily of genes.

Sample Type	Methylated (%)			
	*TOX*	*TOX2*	*TOX3*	*TOX4*
**Normal tissue**				
NHBEC	0/20 (0)	0/20 (0)	0/20 (0)	0/20 (0)
HBEC	0/5 (0)	0/5 (0)	0/5 (0)	0/5 (0)
PBMC	0/10 (0)	0/10 (0)	0/10 (0)	0/10 (0)
DNLT	0/8 (0)	0/8 (0)	0/8 (0)	0/8 (0)
**Breast cancer**				
Cell lines	3/4 (75)	3/4 (75)	2/4 (50)	0/4 (0)
Primary tumors	34/80 (43)^A^	18/80 (23)	24/80 (30)^A^	ND
**Lung cancer**				
Cell lines	4/20 (20)	4/20 (20)	5/20 (25)	0/20 (0)
Primary tumors	9/190 (5)	54/190 (28)	110/190 (58)	ND
**Adenocarcinoma**	7/171 (4)	51/171 (30)	95/171 (56)	ND
Current smokers	1/37 (3)	16/37 (43)^B^	18/37 (49)	ND
Former smokers	4/59 (7)	17/59 (29)	29/59 (49)	ND
Never smokers	2/75 (3)	18/75 (24)	48/75 (64)^C^	ND
**Squamous cell carcinoma**	2/19 (11)	3/19 (16)	15/19 (79)^D^	ND

A Methylation of *TOX* was significantly more prevalent in breast than lung tumor (p<0.001). In contrast, *TOX3* methylation was more common in lung than breast tumor (p<0.001).

B Among NSCLC patients, the prevalence for *TOX2* methylation in current smokers was significantly higher than never smokers (p<0.05) as well as current non-smokers (former and never smokers combined) (p<0.05).

C *TOX3* methylation in primary lung tumors was marginally more prevalent in never smokers compared to current or former smokers (p = 0.05).

D *TOX3* methylation in primary lung tumors was more prevalent in squamous cell carcinoma compared to adenocarcinoma (p = 0.05).

### Novel *TOX2* transcripts identified

**Figure 2:**
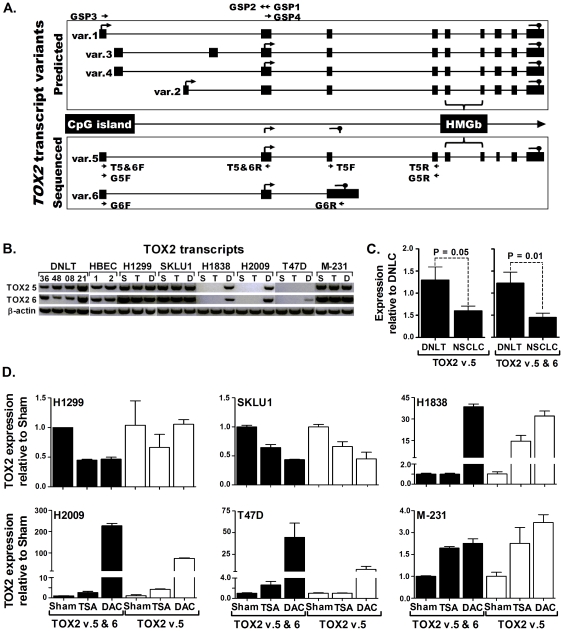
*TOX2* expression in normal and cancer cells. (**A**) Genomic structure of *TOX2*. Top box: Predicted transcript variants of *TOX2* (var.1-4) currently used as reference sequence for *Homo sapiens* chromosome 20, GRCh37.p2, (GenBank accession number NC_000020.10). Bottom box: Transcripts sequenced from human cells (var.5 and 6). Small arrows indicate the location and direction of primer binding sites; T#F or T#R (forward or reverse primers for TaqMan assays) and G#F or G#R (forward or reverse primers for gel-based assays). (**B**) Expression of *TOX2* transcript variants 5 and 6 and the house keeping gene beta-actin in distant normal lung tissue (DNLT), HBEC, and various lung and breast cancer cell lines. In Vehicle-treated (S, for sham) lung cancer (H1838, H2009) and breast cancer (T47D) cell lines with methylated promoter CpG island, both transcripts were silenced and expression of both was primarily restored with 5-Aza-2′-deoxycytidne (D) but not trichostatin A (T) treatment. (**C and D**) TaqMan assays that use distinct primer sets from those used for gel-based assays confirmed results shown in Figure 2B. (**C**) Expression of TOX2 var.5 or both (var.5 & 6) in lung tumors (n = 20) relative to DNLT (n = 10) obtained from NSCLC patients. (**D**) Expression of *TOX2* var.5 or both (var.5+6) in TSA or DAC treated lung and breast cancer cell lines relative to Vehicle-treated (Sham) cell lines.

The primary reference sequence for *Homo sapiens* chromosome 20, GRCh37.p2, (accession number NC_000020.10) predicted (based on automated computational gene prediction methods) four protein coding *TOX2* transcripts [38], [39]. To define the transcripts expressed in lung and breast tissue, 5′ RACE using a set of nested-antisense PCR primers (GSP1 and GSP2) complimentary to regions within exon-2 (present in all four predicted transcripts) was applied (Figure 2A). Following two rounds of amplification, a single 165 bp fragment was generated, cloned, and sequenced. Analysis of five clones revealed that the sequence was similar to the first two exons (exon 1 and 2) of the predicted *TOX2* var.1 (GenBank accession number NM_001098797.1). The remaining three transcript variants predicted to comprise exons 1a (var.4), 1b (var.2), or 1a and 1c (var.3), GenBank accession numbers NM_001098798.1, NM_032883.2, and NM_001098796.1, respectively, were not detected (Figure 2A). The complete sequence of the transcripts were determined through 3′ RACE using a second set of nested-PCR primers complimentary to regions in exon 1 (GSP3) and exon 2 (GSP4). The 3′ RACE produced two transcripts that were confirmed by sequencing and RT-PCR. The longer (2314 bp) of these transcripts (designated *TOX2* var.5) was similar, except at exon 7, to *TOX2* var.1. Exon 7 in var.1 was predicated to have 396 bp sequence. But in *TOX2* var.5, only the 5′ half (198 bp) of this exon was spliced to exon 8, indicating a novel transcript variant distinct from var.1 (Figure 2A). The second transcript (designated *TOX2* var.6) was 1213 bp and consists of three exons (exon 1, 2, and 3). While exon 1 and 2 were similar to transcript var.5, exon 3 was extended further by an additional 752 bp including a stop codon at nucleotides 289–291. In contrast to var.5 as well as any of the four predicted transcripts, *TOX2* var.6 lacks exons 4–9 including the sequences within exons 5 and 6 that encode for the characteristic DNA-binding HMG-box domain (Figure 2A). The complete sequences of the two novel transcripts have been deposited at GenBank [accession numbers JN655166 (TOX2 var.5) and JN655167 (Tox2 var.6)] and are shown in supporting Figures S1A and B.

### *TOX2* methylation is associated with gene silencing

Both *TOX2* transcript variants (var.5 and var.6) were expressed in DNLT and normal bronchial epithelial cells (first 6 lanes of Figure 2B). Quantitative analysis of these transcripts in paired tumor-normal tissues obtained from NSCLC patients revealed that both transcripts were significantly reduced in lung tumors compared to normal lung (Figure 2C). The relationship between hypermethylation of *TOX2* promoter and expression of the two transcripts was compared in normal and malignant cell lines. In HBEC and the lung cancer cell lines *H1299* and *SKLU1* with unmethylated *TOX2* promoter CpG island (Figures 1A and 1B), both *TOX2* transcripts were expressed at levels similar to DNLT (Figure 2B). In contrast, both transcripts were silenced in lung (*H1838* and *H2009*) and breast (*T47D*) cancer cell lines in conjunction with densely methylated promoter CpG islands. In *MDA-MB-231 (M-231)*, the presence of some undigested PCR products in the COBRA assay (Figure 1A) and the absence of methylation in 2 out of 5 bisulfite sequenced clones (nearly all 51 CpGs in clones 1 and 5 are unmethylated, Figure 1B) indicate that the *TOX2* promoter is hemi-methylated in this cell line. In agreement with this, both transcripts of *TOX2* were expressed in *MDA-MB-231* (Figure 2B).

### DAC treatment restores *TOX2* expression

Lung and breast cancer-derived cell lines with or without *TOX2* promoter hypermethylation were treated with Vehicle (S for sham), the DNA demethylating agent 5-Aza-2′-deoxycytidne (DAC), or the histone deacetylase inhibitor trichostatin A (TSA) as described to evaluate the contribution of cytosine methylation and chromatin remodeling in silencing this gene. Expression of both *TOX2* transcripts could be restored in *H1838* and *H2009* after DAC (D) treatment (Figure 2B). DAC treatment could only partially restore expression of transcript var.6 but not var.5 in *T47D*. Quantitative analysis of these transcripts using TaqMan primer-probe sets that are distinct from the primers used for the gel-based assays confirmed these findings and showed that DAC treatment led to 39–227- and 7–73-fold increased expression of both transcripts or var. 5 alone, respectively (Figure 2D). TSA treatment had little or no effect on the expression of these transcripts (Figure 2B and 2D). In cell lines with unmethylated or a hemi-methylated *TOX2* promoter, both transcripts were detected in vehicle treated cells and treatment with either TSA or DAC had little or no effect on the expression of these transcripts (Figure 2B and 2D).

### Distinct methylation of *TOX* subfamily genes between lung and breast tumors

**Figure 3:**
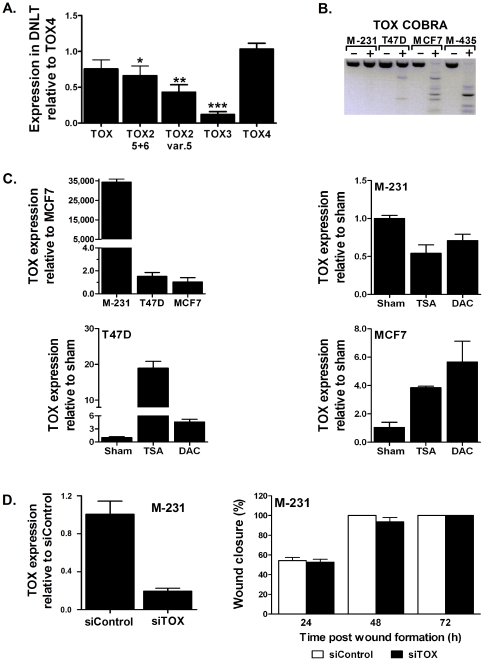
Relative expression of TOX subfamily genes in normal lung tissue. (**A**) Expression of each gene was quantified using TaqMan assays and the level of *TOX4*, which is unmethylated in all samples and expressed the highest in normal lung tissue, was used as a reference to calculate the relative level of the remaining genes. * p = 0.03, ** p<0.001, *** p<0.0001 compared to *TOX4*. (**B**) COBRA conducted as described for Figure 1A. (**C**) *TOX* expression was measured relative to its expression in *MCF-7* (Top left) or vehicle treated *MDA-MB-231 (M-231)*, *T47D*, or *MCF-7*. (**D**) Transfection of *M-231* with siTOX reduced its expression by 75% compared to siControl (left) but this did not alter the migration potential of the cells.

The *TOX* subfamily in human includes three additional members (TOX, TOX3, and TOX4) that share similar genomic structure with *TOX2* including conserved intron/exon boundaries, high protein homology, and a similarly located promoter CpG island (Table S3). Thus, these genes are considered to arise through gene duplication [16]. The DNA-binding HMG-box motif of TOX2 is nearly identical (92, 94, and 94% homology) to that of TOX, TOX3, and TOX4, and overall the three proteins, respectively share 59, 65, and 62% amino acid homology to TOX2. These similarities and the discovery of aberrant methylation of *TOX2* in lung and breast tumors prompted us to evaluate the methylation status of the remaining *TOX* subfamily genes. The promoter CpG islands of *TOX* and *TOX3* were also methylated in 20 and 25% lung, and 75 and 50% breast cancers cell lines, respectively (Table 1). In contrast, the promoter CpG island of TOX4 was unmethylated in all lung and breast cancer cell lines, and none of these genes were methylated in normal tissue (NHBEC, HBEC, PBMC, and DNLT). Among primary tumors, *TOX* and *TOX3* were methylated in 5% (9/190) and 58% (110/190) lung and 43% (34/80) and 30% (24/80) breast tumors, respectively (Table 1). Interestingly, the prevalence for *TOX3* methylation among lung cancer patients was significantly greater in squamous cell carcinoma 79% (15/19) compared to adenocarcinoma 56% (95/171). The level of expression of *TOX* subfamily genes in DNLT were inversely correlated with the prevalence for methylation of primary tumors (Figure 3A). Compared to *TOX4*, expression of *TOX*, *TOX2*, and *TOX3* in normal tissue was reduced by 25, 44, and 88%, respectively.

Tumor-specific hypermethylation of *TOX* in breast tumors but not in the adjacent normal tissue has been recently demonstrated as a potential novel tumor biomarker [10]. Our data revealed that *TOX* is hypermethylated in 43% of breast tumors and further studies demonstrated that expression of this gene in breast cancer cells is epigenetically silenced. *TOX* is completely (*MCF-7* and *MDA-MB-435*) or partially (*T47D*) methylated in three out of four breast cancer cell lines (Figure 3B). As shown in Figure 3C (top left) expression of *TOX* in the methylated cell lines (including the weakly methylated *T47D*) is dramatically reduced compared to the unmethylated cell line (*MDA-MB-231*). Treatment with either TSA or DAC led to partial re-expression of *TOX* in the methylated breast cancer cell lines (Figure 3C). Consistent with the minor methylation seen in *T47D* (Figure 3B), treatment with TSA resulted in ∼20-fold increased expression, more than the ∼5-fold increase seen after DAC treatment (Figure 3C, bottom left). To evaluate the impact of methylation-mediated silencing of *TOX* in breast cancer, *MDA-MB-231* cells where the gene is unmethylated and abundantly expressed were transfected with control (siControl) or *TOX* specific (siTOX) siRNAs. Although *TOX* expression in siTOX transfected cells was reduced by 75% compared to the siControl, it did not significantly affect the proliferation (measured by MTT, not shown) or migration potential of the cells (Figure 3D).

### *TOX3* is silenced by promoter hypermethylation

**Figure 4:**
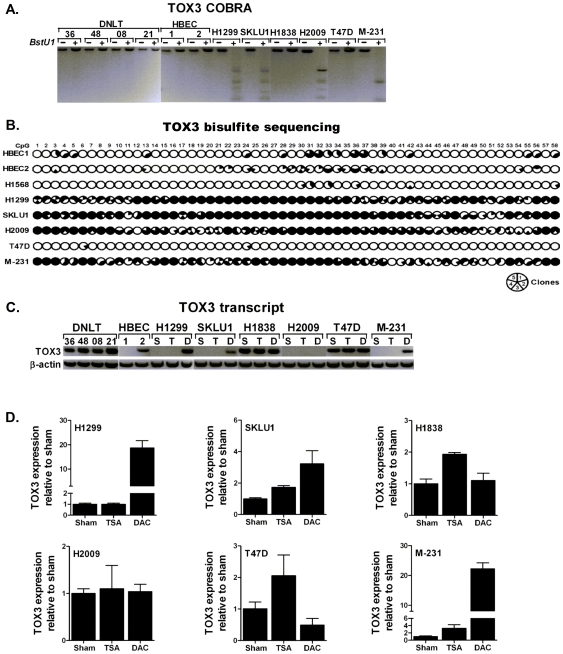
Methylation and expression of *TOX3* in lung and breast cancer. (**A**) COBRA and (**B**) bisulfite sequencing assays were used to assess the methylation status of *TOX3* and the results are summarized as described for figure 1. (**C**) Expression of *TOX3* and beta-actin in DNLT, HBEC, and various lung and breast cancer cell lines. *TOX3* was silenced in vehicle-treated (S) lung cancer (H1199, SKLU1, and H2009) and breast cancer (MDA-MB-231, abbreviated as M-231) cell lines with methylated promoter CpG island. Treatment with 5-Aza-2′-deoxycytidne (D) but not trichostatin A (T) restored *TOX3* expression. (**D**) Quantitative analysis of *TOX3* in lung and breast cancer cell lines treated with Vehicle (Sham), TSA, and DAC using a TaqMan assay that uses primer sets distinct from the primers used for gel-based assays confirmed results shown in figure 3C.

The level and distribution of methylation across the *TOX3* promoter CpG island and its impact on the expression of this gene was evaluated as described for *TOX2*. COBRA (Figure 4A) and bisulfite sequencing of 58 CpGs within the *TOX3* promoter CpG island (Figure 4B) revealed that *TOX3* is unmethylated in all normal samples and some lung and breast cancer cell lines. However, these assays also revealed dense methylation of *TOX3* promoter in some lung and breast cancer cell lines. With the exception of HBEC1, *TOX3* expression was readily detected in all unmethylated samples including normal lung tissue, HBEC2, as well as lung and breast cancer cell lines with unmethylated *TOX3* promoter such as *H1838* and *T47D* (Figure 4C). In contrast, *TOX3* expression was completely silenced in sham (S) lung and breast cancer cell lines with dense promoter methylation such as *H1299*, *SKLU1*, *H2009*, and *MDA-MB-231* (Figures 4A–C). With the exception of H2009, *TOX3* expression in the methylated cell lines was mostly restored after DAC treatment (Figure 4C). Quantitative TaqMan assays using primer probes distinct from those used for the gel-based assays also reproduced the observed re-expression of *TOX3* after DAC treatment (Figure 4D).

### Epigenetic inactivation of *TOX2* and *TOX3* modulates multiple genes

**Figure 5:**
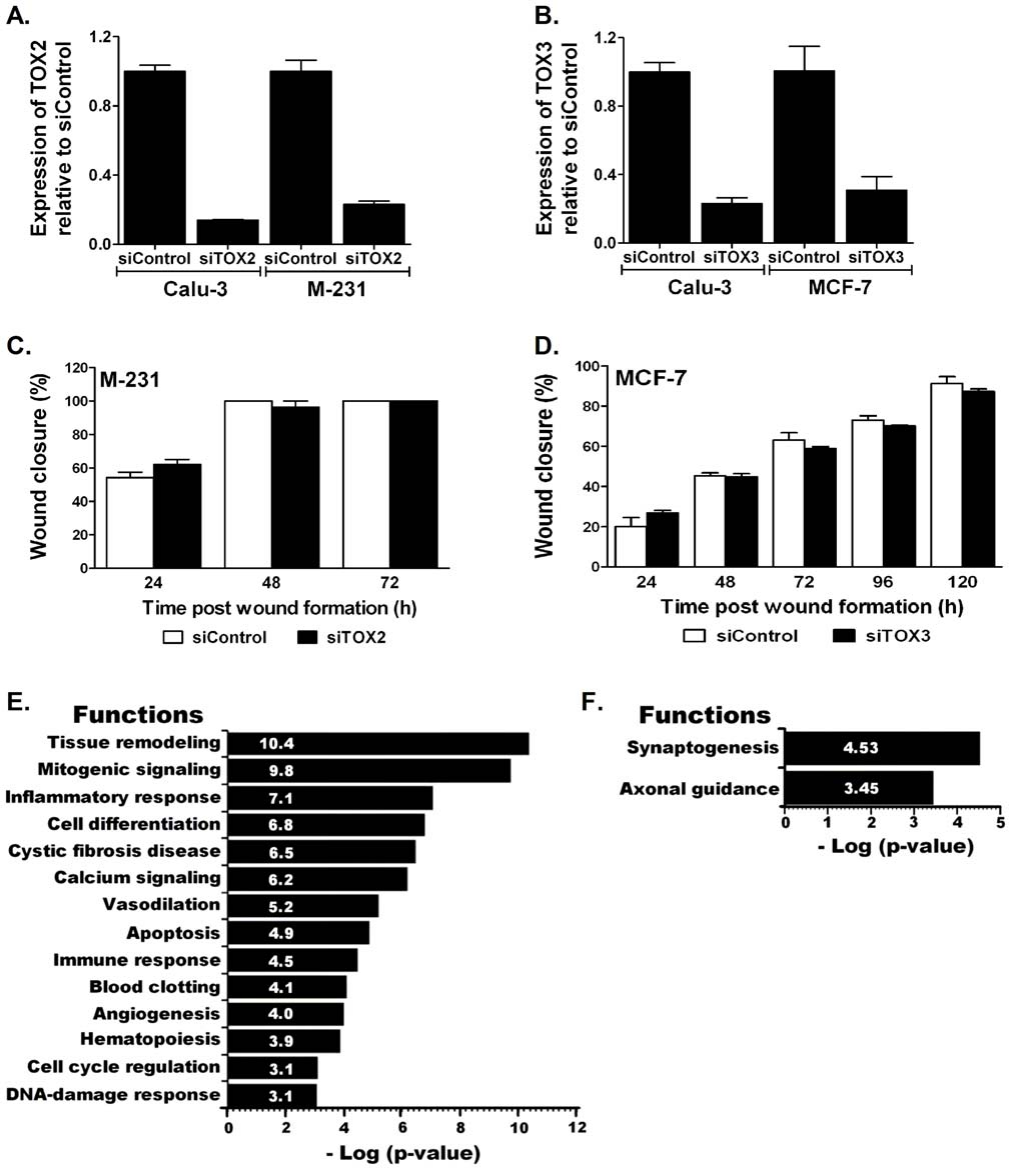
Genome-wide impact of epigenetic inactivation of *TOX2* and *TOX3*. Transfection of (**A**) *Calu-3* and *MDA-MB-231 (M-231)* with siRNAs targeting *TOX2* (*siTOX2*) or (**B**) *Calu-3* and *MCF-7* targeting *TOX3* (*siTOX3*) reduced expression of these genes by 70–86% compared to cells transfected with control siRNA (siControl). (**C and D**) However, knockdown of these genes did not change the migration potential of these cells. Genome-wide gene expression assays comparing *Calu-3* cells transfected with (**E**) siControl vs. siTOX2 or (**F**) siControl vs. siTOX3 revealed genes and pathways modulated by epigenetic inactivation of these genes.

The impact of promoter methylation-mediated silencing of *TOX2* and *TOX3* was similarly investigated *in vitro* using siRNAs targeting the two genes. Lung (*Calu-3* for both genes) and breast cancer cell lines (*MDA-MB-231* for *TOX2* and *MCF-7* for *TOX3*) where the two genes are expressed were selected for transfection. Quantitative TaqMan assays confirmed that compared to control siRNA (siControl), transfections with siTOX2 and siTOX3 reduced expression of *TOX2* (both transcripts) and *TOX3* by 70–86% (Figure 5A and 5B). Similar to *TOX*, MTT and wound closure assays revealed that knock down of either of these genes did not affect the proliferation (not shown) or migration potential of lung or breast cancer cells (Figure 5C and 5D).

The potential impact of epigenetic inactivation of *TOX2* and *TOX3* on genes and pathways across the genome was evaluated using a genome-wide transcriptome array conducted on *Calu-3* cells transfected with siControl, siTOX2, or siTOX3. This genome-wide expression array revealed knockdown of *TOX2* affected the expression of 830 genes (437 increased and 393 decreased) by more than 1.5-fold and significantly modified multiple pathways including tissue remodeling, mitogenic signaling, inflammatory and immune responses, apoptosis, cell cycle regulation and differentiation, and multiple regulatory pathways of the circulatory system (Figure 5E). Genes that showed ≥2-fold increased (73) or decreased (71) expression after *TOX2* knockdown are listed in supporting Table S4 and S5. Despite affecting the expression of hundreds of genes and modulating multiple pathways, *TOX2* knockdown did not impact cell proliferation, cell death, cell migration or growth in soft agar of Calu-3 cells (not shown). In contrast to the broad pathways modulated by *TOX2* inactivation, *TOX3* knockdown resulted in more specific changes targeting genes involved in neuronal development. Overall, *TOX3* knockdown affected the expression of 275 genes by ≥1.5 fold (50 increased and 225 decreased) and significantly modulated synaptogenesis and axonal guidance pathways (Figure 5F). Genes with ≥2-fold increased (7) or decreased (27) expression after siRNA-mediated *TOX3* silencing are shown in Table S6. Similar to *TOX2*, *TOX3* knockdown also did not significantly altered cell proliferation, cell death, or migration properties of Calu-3 cells (not shown).

## Discussion

This study identified for the first time aberrant hypermethylation of the *TOX2* promoter CpG island in cancer and characterized its potential contribution to carcinogenesis. Two novel transcripts of *TOX2* that are distinct from variants predicted for this gene were cloned and sequenced. Dense methylation of the *TOX2* promoter silenced both of these transcripts in lung and breast cancer cells and DAC treatment restored expression of both transcripts *in vitro* confirming epigenetic regulation. Expression of both transcripts was significantly lower in primary lung tumors compared to distant normal lung tissue. Extension of these assays to other members of *TOX* subfamily genes that share similar genomic structure and protein homology to TOX2 revealed distinct methylation profiles between lung and breast tumors. Methylation of *TOX* was almost exclusively seen in breast cancer, whereas *TOX3* methylation was more prevalent in lung than breast cancer. Furthermore, the genome-wide impact of epigenetic inactivation assessed by siRNA revealed that *TOX2* knockdown modulated multiple molecular pathways including important modulators of tumor microenvironment such as tissue remodeling, inflammatory response, and cell differentiation. In contrast, *TOX3* knockdown specifically targeted pathways involved in neuronal development and axonal guidance, recently defined functions of TOX3 [22], [23]. Although knockdown of either gene by siRNA did not alter cell proliferation, survival, or migration significantly, the differential methylation profile of TOX subfamily genes across tumor type and histology and the genes and pathways affected by epigenetic silencing of these genes could be exploited for developing tumor-type specific biomarkers [10], [40].

The four transcript variants currently used as the primary reference sequence for *TOX2* in *Homo sapiens* are predicted sequences generated through automated computational gene prediction methods [38], [39]. This study provides the first experimentally generated transcripts of *TOX2* that were cloned and sequenced from normal and malignant lung and breast tissue. The amino acid sequence deduced from one of these transcripts, *TOX2* var.5, is over 93% homologous to the granulosa cell HMG-box protein 1 (GCX-1), an ortholog of TOX2 identified from a rat granulosa cell cDNA library [21]. GCX-1 is a potent transcriptional activator exclusively expressed in the hypothalamo-pituitary-gonadal axis of Wistar rats, and functions as a specific regulator of follicular development and other events related to reproduction [21]. The perfect homology (100%) between the HMG-box domains of TOX2 var.5 (human) and GCX-1 (rat) indicates this gene is highly conserved across the two species and suggests that the protein encoded by *TOX2* var.5 in humans may similarly function as a transcription factor [21]. In contrast, *TOX2* var.6 does not encode for the characteristic DNA binding HMG-box domain suggesting that the protein encoded by this variant may not directly bind to DNA and potentially could serve as a negative competitor to the remaining *TOX2* variants. Expression of these transcripts in normal lung suggests TOX2 may have different or additional functions in humans, thus further studies are required to define the tissue distribution and specific roles of these TOX2 variants.

It is now well established that cancer cells accumulate aberrant methylation of hundreds of genes [41]. Emerging evidence from genome-wide and candidate gene methylation studies indicate that the methylation profile of some of these genes could discriminate tumors by phenotypes such as cancer-type, histology, and stage of disease [10], [40]. In this regard, the differential methylation of *TOX* and *TOX3* between lung and breast cancer, *TOX2* across cigarette smoking habit, and *TOX3* by the histology of lung cancer suggest, methylation of *TOX* subfamily genes could be important biomarkers for cancer profiling. In agreement with our findings, a recent study after evaluation of leukemia and various solid tumors including prostate, colorectal, breast, and liver cancers, also identified *TOX* methylation as important biomarker specifically for breast cancer [10]. Interestingly, this study also used the MCA/RDA assay to identify methylation of *TOX*, among other genes, and reported exactly similar methylation of *TOX* in 3/4 (75%) breast cancer cell lines (3 of the cell lines are different from those used in our study) and 10/24 (42%) in primary breast tumors.

Although the cause of differential methylation patterns among cancer types is not well defined, different susceptibility of CpG islands for methylation, which also varies by cell/tissue type, is considered to play a major role. Not all CpG islands are equally susceptible for methylation during carcinogenesis and one of the prominent factors associated with susceptibility/resistance of promoter CpG island methylation is the level of promoter activity. CpG islands in the promoter region of housekeeping and other constantly expressed genes are often protected from methylation. In contrast, CpG islands within non-coding sequences, repetitive elements, and promoter regions of tissue specific genes with lower gene activity are prone to methylation [42]. Although direct evidence linking transcription factor binding to promoter region with protection from methylation is yet to be established, the role of transient reduction in gene expression as a trigger for methylation has been demonstrated [43]. In agreement with this supposition, the level of expression of *TOX* subfamily genes in normal lung was inversely related to the prevalence for methylation in lung tumors. *TOX3*, which has the most commonly methylated promoter CpG island in lung tumors (58%) was expressed at the lowest level in normal lung. Conversely, expression of *TOX4*, which is unmethylated in all samples analyzed, was the highest in normal lung. The fact that the expression patterns and functions of *TOX* subfamily genes vary across tissue types also support different susceptibility to methylation leading to tumor-specific methylation profile.

TOX is highly expressed in the thymus and plays a critical role in T-cell selection, differentiation, and maturation [20]. Whereas TOX3 is a neuronal survival factor that regulates Ca^2+^-dependent transcription in neurons [22], [23]. Consistent with this function, our data also revealed that epigenetic inactivation of *TOX3* specifically modulates pathways involved in synaptogenesis and axonal guidance. A recent study revealed that TOX4 is recruited to DNA-damage specifically induced by platinum compounds (cisplatin and oxaliplatin but not UV) indicating a potential role in DNA damage and repair [24]. Currently, there is no data regarding the normal function of TOX2. GCX-1, the rat ortholog of TOX2, is a potent transcriptional activator involved in the hypothalamo-pitutary-gonadal axis of reproduction [21]. However, due to a specified approach of the study, the expression and function of GCX-1 in rat lung and mammary tissue is unclear. Our data show that TOX2 is expressed in human lungs and epigenetic inactivation of this gene in lung cancer modulates multiple pathways. Among these pathways the involvement of tissue remodeling, inflammatory response, cell differentiation, apoptosis, cell cycle regulation, and DNA-damage response in carcinogenesis is well established, substantiating a role for TOX2 in contributing to early malignant changes and modulation of the tumor microenvironment *in vivo*.

## Supporting Information

Figure S1 **Sequences of** ***TOX2*** **transcript variants identified in this study.** Two novel transcript variants of *TOX2* that are unique from any of the predicted transcripts were identified. (**A**) cDNA sequence of transcript variant 5 is largely similar to the predicted *TOX2* var.1 (Accession number NM_001098797.1) except the 3′ half (186 bp) of exon-7 is missing in var.5. A sequence variation at position 140, where a T nucleotide was missing in *TOX2* var.5 was also found. (**B**) cDNA sequence of the second novel transcript variant (*TOX2* var.6) identified in this study was similar to *TOX2* var.5 up to exon-3, including the single nucleotide variation at position 140. However, exon-3 was extended further by 754 nucleotides including a stop codon at nucleotide positions 289–291 bp. The sequence of this additional component of exon-3 was similar (other than a C to T variation at nucleotide 1047) to the genomic sequence of *TOX2* (Accession number NC_000020.10). Sequence variations seen in the two novel transcripts (a TT instead of TTT in var.5 and a C to T in var.5) are highlighted, and translation start and stop codons are shown in bold and underlined. These two nucleotide sequences are deposited at GenBank and have been provided GenBank accession numbers JN655166 for TOX2 var.5 and JN655167 for Tox2 var.6.
(DOC)

Table S1 **Primer sequences and amplification conditions for methylation and expression assays.**
(DOC)

Table S2 **Primer sequences and amplification conditions used for RACE.**
(DOC)

Table S3 **Characteristics of TOX high mobility group box family members.**
(DOC)

Table S4 **Genes with ≥2-fold increase as a result of TOX2 knockdown.**
(DOC)

Table S5 **Genes with ≥2-fold decrease as a result of TOX2 knockdown.**
(DOC)

Table S6 **Genes with ≥2-fold change as a result of TOX3 knockdown.**
(DOC)
