# Supplementary material for: Differential Epigenetic Regulation of TOX Subfamily High Mobility Group Box Genes in Lung and Breast Cancers
Source: PLoS One. 2012 Apr 4;7(4):e34850. doi: 10.1371/journal.pone.0034850 (PMC3319602; doi:10.1371/journal.pone.0034850)
Supplement: Figure S1 — Sequences of TOX2 transcript variants identified in this study. Two novel transcript variants of TOX2 that are unique from any of the predicted transcripts were identified. (A) cDNA sequence of transcript variant 5 is largely similar to the predicted TOX2 var.1 (Accession number NM_001098797.1) except the 3′ half (186 bp) of exon-7 is missing in var.5. A sequence variation at position 140, where a T nucleotide was missing in TOX2 var.5 was also found. (B) cDNA sequence of the second novel transcript variant (TOX2 var.6) identified in this study was similar to TOX2 var.5 up to exon-3, including the single nucleotide variation at position 140. However, exon-3 was extended further by 754 nucleotides including a stop codon at nucleotide positions 289–291 bp. The sequence of this additional component of exon-3 was similar (other than a C to T variation at nucleotide 1047) to the genomic sequence of TOX2 (Accession number NC_000020.10). Sequence variations seen in the two novel transcripts (a TT instead of TTT in var.5 and a C to T in var.5) are highlighted, and translation start and stop codons are shown in bold and underlined. These two nucleotide sequences are deposited at GenBank and have been provided GenBank accession numbers JN655166 for TOX2 var.5 and JN655167 for Tox2 var.6. (DOC) [file pone.0034850.s001.doc]

**Figure S1**

**A.**

ACTGCCCGCG GGAGCCGCCG CCGCCGCCGC CGCGCCCGCC ATGGACGTCC GCCTGTACCC CTCGGCGCCC GCGGTGGGCG CGCGGCCCGG GGCCGAGCCG GCCGGCCTGG CGCACCTGGA CTATTACCAC GGCGGCAAGT TGATGGTGAC AGTGCCTACG TGGGG**ATG**AG TGACGGAAAC CCAGAGCTCC TGTCAACCAG CCAGACCTAC AACGGCCAGA GCGAGAACAA CGAAGACTAT GAGATCCCCC CGATAACACC TCCCAACCTC CCGGAGCCAT CCCTCCTGCA CCTGGGGGAC CACGAAGCCA GCTACCACTC GCTGTGCCAC GGCCTCACCC CCAACGGTCT GCTCCCTGCC TACTCCTATC AGGCCATGGA CCTCCCAGCC ATCATGGTGT CCAACATGCT AGCACAGGAC AGCCACCTGC TGTCGGGCCA GCTGCCCACG ATCCAGGAGA TGGTCCACTC GGAAGTGGCT GCCTATGACT CGGGCCGGCC CGGGCCCCTG CTGGGTCGCC CGGCAATGCT GGCCAGCCAC ATGAGTGCCC TCAGCCAGTC CCAGCTCATC TCGCAGATGG GCATCCGGAG CAGCATCGCC CACAGCTCCC CATCACCGCC GGGGAGCAAG TCAGCGACCC CCTCTCCCTC CAGCTCCACT CAGGAAGAGG AGTCGGAAGT GCATTTCAAG ATCTCGGGAG AAAAGAGACC TTCAGCCGAC CCAGGAAAAA AGGCCAAGAA CCCGAAGAAG AAGAAAAAGA AGGACCCCAA TGAGCCGCAG AAGCCTGTGT CGGCCTACGC ACTCTTCTTC AGAGACACTC AGGCCGCCAT CAAGGGTCAG AACCCCAGTG CCACTTTCGG TGACGTGTCC AAAATCGTGG CCTCCATGTG GGACAGCCTG GGAGAGGAAC AGAAGCAGGC CTACAAGAGG AAGACAGAAG CAGCAAAGAA GGAATATCTG AAGGCCCTGG CAGCCTACCG GGCTAGCCTC GTCTCCAAGA GCTCCCCAGA TCAAGGTGAG ACCAAGAGCA CTCAGGCAAA CCCACCAGCC AAAATGCTCC CACCCAAGCA GCCCATGTAT GCCATGCCAG GCCTGGCCTC CTTCCTGACG CCGTCGGACC TGCAGGCCTT CCGCAGTGGG GCCTCCCCTG CCAGCCTCGC CCGGACGCTG GGCTCCAAGT CTCTGCTGCC AGGCCTCAGA CTTCCCGCAC ATCTC**TGA**GT TCCCCAGCAG CTCGGGATCC TGCTCACCTG GCCCATCCAA CCCCACCAGC AGCGGGGACT GGGACAGCAG CTACCCCAGT GGGGAGTGTG GCATCAGCAC CTGCAGCCTG CTCCCCAGGG ACAAATCGCT CTACCTCACC TAATCCCGCC TCCCTACCAT CCCTGAGGCT CGCTGGAAGG CACTGCTCAG AGCCTGAAGG GCTGACAGCA GAAAAGAGGC CCTGGCCAGA GGCAGGGTGG CCCATCGGAG AGAGCAGTGA CACACCCATT GCCCGGGGGC TGAGTCTCTT CCTCAACCTC CCACCAGACT CTGCAGAGGC AGCCCACTGC CCACCACCAG CCCAAAGAAC CTGCAGGAAC CTTCCGCCCG CTGACCTGCT TGCTCCAGGG TAACTGTGGA CCCTGTCCTC GCCCTGCGCA CGGTACCCTA TGTCTGGACA CCCGGCCCCA GCTCCAGCCC CAGCCCAGGT GGGCCGCCCC TGGCGGGGTC GCTTACCAAC GGACACCCAC CCCAGATGCA TGGGCCAGAG GGCCGGCCCC CGGCATAGAT GTGCACATCG GTTTTCCAGT GTGAACAAAA GATTACGAAA CCTAGAAACT GTTGGTTCCG TGTAAGTAGT TGACTACGTG TTTTAGAACT GTGCTGAAGA CATCTGTAAG ACTATTTTGT GGGGGAAAAA AGTAGTTTCC TTTAAGGTAA AAAGCATTTT ATATGATCCT TAGCACATTT TTAAGTTTTA TCTTAAGGGA GACGCGCACA AAAGCGGCTG CCAAACCGTT TCGTCATCCT CACAGCAAGG ACCGGACGCT TGCTAGCCAC CCCGGAGCAC TGCTCTCCTT TTAATCATGT ATTCATCTAT TTTAAATTGC CGGCGACGAC TTTTGTCTAT TTATGAAGAA ACCTTGAGAA CGAAGTTACA GCTTATCCTA CCGTGTGTGT GGTTTTGGGG TTTCGTTTGG GTTTGGGTTC TTGACGTCGT TTGCAGCTGT TTCCTGGCCC TGGCGAGTGT CTGTCTTGGT GCCCAGTGCT TCTCTCAAAT CTCTTTATAA TAAAACTTCT GAAAAGCTGA AAAAAAAAAA AAAAAAAAAA AAAA

**B.**

ACTGCCCGCG GGAGCCGCCG CCGCCGCCGC CGCGCCCGCC ATGGACGTCC GCCTGTACCC CTCGGCGCCC GCGGTGGGCG CGCGGCCCGG GGCCGAGCCG GCCGGCCTGG CGCACCTGGA CTATTACCAC GGCGGCAAGT TGATGGTGAC AGTGCCTACG TGGGG**ATG**AG TGACGGAAAC CCAGAGCTCC TGTCAACCAG CCAGACCTAC AACGGCCAGA GCGAGAACAA CGAAGACTAT GAGATCCCCC CGATAACACC TCCCAACCTC CCGGAGCCAT CCCTCCTGCA CCTGGGGGAC CACGAAGCCA GCTACCACTC GCTGTGCCAC GGCCTCACCC CCAACGGTCT GCTCCCTGCC TACTCCTATC AGGCCATGGA CCTCCCAGCC ATCATGGTGT CCAACATGCT AGCACAGGAC AGCCACCTGC TGTCGGGCCA GCTGCCCACG GTGAGTCCCT ATCGCCTGCT GCAGTTCCTG CTGATGACAG CAGGGAGGGG GTTGAGAGGG AAGCAGAAGA ATGTTGATGC TTTGATAAGA ACTCTCTGCT CATGGGCAGG GTCTGGGTTT ACCTGGTTGC TTGGAGTTGG TAATCCCATG CTGGGATTAC CTGGTAGCAT GGGAGTCTGT GGGACACGGT GGGTGGGCAT GGAGAGAAAT CTAATATCAG CACACTGCTT CCTTCTGGAG CTCTGCGTAC AGTATCTCGG TATTGGTTTG CATTATTTGT TAATAGTA**TA A**TCTCATTTT TATTTTTTAA CAGTTTTATT GAGGTATAAT TTATATTCCA TACAATTCAC CCATTTAAAG TATACTACTT AATGACTTTT CATATATTGA CAGAGTTGTG TGACCATTAA CACGATTGAT TTTAGAACAT TTCATCACCC CATAAAGAAA CACTAGCTAG GCACAGTGTT GTGTACCTAT AATCTCAGCT ACTTGGGAGG CTAAATGGGA GGATCGCTTG AGCCCAGGAG TCCTGGGCTG TAGTGCCCTA TGCAAATCAG TTGGCTGCAC TAGCTTTGGC ATCAGTATGG TGACCCCCTG GGAGAGGGGA ACCACTAGGT TGCTTAAGGA GGGGTAAACT GGCCACGGTC GGAAACAGAG CAGGTCAAAA CTCCCATGCT AACCAGTAGT GGGATTGTGC CTGTGAAAAG GCACTGCACT CCGGGCATCA TGGCAAGACC CTATCACGAA AAAAAAAAAA AAA
