# Supplementary material for: Differential Epigenetic Regulation of TOX Subfamily High Mobility Group Box Genes in Lung and Breast Cancers
Source: PLoS One. 2012 Apr 4;7(4):e34850. doi: 10.1371/journal.pone.0034850 (PMC3319602; doi:10.1371/journal.pone.0034850)
Supplement: Table S1 — Primer sequences and amplification conditions for methylation and expression assays. (DOC) [file pone.0034850.s002.doc]

**Table S1: Primer sequences and amplification conditions for methylation and expression assays**

| **Primers ^A^** | **Primers** | | **PCR conditions ^B^** | | |
| --- | --- | --- | --- | --- | --- |
|  | **Forward** | **Reverse** | **Annealing** | | **Size (bp)** |
|  |  |  | **T (°C)** | **t (s)** |  |
| **COBRA** |  |  |  |  |  |
| TOX | GAGTTGTTTGATGTGAGAGTG | CTCTTACTCTACCTCCRTTC | 57 | 60 | 486 |
| TOX2 | GTTTAGTTYGGAGGAAGGATTTTTA | TAATAATAATCCAAATACRCCAAACC | 60 | 60 | 331 |
| TOX3 | TTAGTTTYGTTTAGYGTATTTG | ATAAAACCTCACATCCATACC | 54 | 60 | 361 |
| TOX4 | GAATTTGATAGTTAAGAAGGTTG | CCTCCCCACTACCCTACC | 54 | 60 | 344 |
| **MSP** |  |  |  |  |  |
| TOX | GTAGTTTTTTTGTGTGCGACGC | GAACGTCCCGAACCGAACG | 63 | 30 | 227 |
| TOX2 | GGTCGGAATAATAGCGCGC | GAACGTCCATAACGAACGCG | 68 | 30 | 181 |
| TOX3 | GATTCGGGTTTTAGCGTCGC | CTAAATCCACCGTCGAAAACG | 64 | 30 | 236 |
| **RT-PCR** |  |  |  |  |  |
| TOX2-5 | CCACGGCGGCAAGTTGATGGTGAC | GCCCGAGTCATAGGCAGCCACTTC | 68 | 30 | 369 |
| TOX2-6 | CCACGGCGGCAAGTTGATGGTGAC | GGAACTGCAGCAGGCGATAGGGAC | 68 | 30 | 354 |
| TOX3 | CCTGCCAGCCTGGACTTC | GAGGAGGCGTGATTGGTGG | 64 | 30 | 181 |
| β-actin | CCAGCCTTCCTTCCTGGGCAT | AGGAGCAATGATCTTGATCTTCATT | 64 | 30 | 211 |

1. Primers used for COBRA were also used for bisulfite sequencing.
2. All PCR done to 40 cycles and *BstU I* enzyme was used to digest PCR products for COBRA.
