# Supplementary material for: Differential Epigenetic Regulation of TOX Subfamily High Mobility Group Box Genes in Lung and Breast Cancers
Source: PLoS One. 2012 Apr 4;7(4):e34850. doi: 10.1371/journal.pone.0034850 (PMC3319602; doi:10.1371/journal.pone.0034850)
Supplement: Table S2 — Primer sequences and amplification conditions used for RACE. (DOC) [file pone.0034850.s003.doc]

**Table S2: Primer sequences and amplification conditions used for RACE.**

|  | **^A^ Primer**  **name** | **Primer sequences**  **(5’ to 3’)** | | | **Step** | | **Temp.**  **(°C)** | **Time** | | **Number**  **of cycles** | |
| --- | --- | --- | --- | --- | --- | --- | --- | --- | --- | --- | --- |
| **5’ RACE** |  |  | | |  | |  |  | |  | |
|  |  |  | | | Initial Den. | | 94 | 2 min | | 1 | |
| **Stage -1** |  |  | | | Den. | | 94 | 30 sec | | 5 | |
|  |  |  | | | Ann./Ext. | | 72 | 1 min | |  |  |
|  | GSP1 | GAGCTCTGGGTTTCCGTCACTCATC | | | Den. | | 94 | 30 sec | | 5 | |
|  |  |  | | | Ann./Ext. | | 70 | 1 min | |  |  |
|  | GRAP | GCACAGAGGACACUGACAUGGACUGA | | | Den. | | 94 | 30 sec | | 25 | |
|  |  |  | | | Final Ann. | | 68 | 1 min | |  |  |
|  |  |  | | | Final Ext. | | 72 | 1 min | | 1 | |
| **Stage-2** |  |  | | | Initial Den. | | 94 | 2 min | | 1 | |
|  | GSP2 | CCCACGTAGGCACTGTCACCATCAA | | | Den. | | 94 | 30 sec | | 25 | |
|  |  |  | | | Ann | | 65 | 30 sec | |  |  |
|  | GRNAP | GGACACTGACATGGACTGAAGGAGTA | | | Ext. | | 68 | 2 min | |  |  |
|  |  |  | | | Final Ext. | | 68 | 10 min | | 1 | |
| **3’ RACE** | | |  |  | |  | | |  | |  |
|  |  |  | | | Initial Den. | | 94 | 2 min | | 1 | |
| **Stage -1** |  |  | | | Den. | | 94 | 30 sec | | 5 | |
|  |  |  | | | Ann./Ext. | | 72 | 1 min | |  |  |
|  | GSP3 | GGCCTGGCGCACCTGGACTATTAC | | | Den. | | 94 | 30 sec | | 5 | |
|  |  |  | | | Ann./Ext. | | 70 | 1 min | |  |  |
|  | GRAP | GCTGTCAACGATACGCTACGTAACG | | | Den. | | 94 | 30 sec | | 25 | |
|  |  |  | | | Final Ann. | | 68 | 1 min | |  |  |
|  |  |  | | | Final Ext. | | 72 | 1 min | | 1 | |
| **Stage-2** |  |  | | | Initial Den. | | 94 | 2 min | | 1 | |
|  | GSP4 | CTACGTGGGGATGAGTGACGGAAAC | | | Den. | | 94 | 30 sec | | 25 | |
|  |  |  | | | Ann | | 65 | 30 sec | |  |  |
|  | GRNAP | CGCTACGTAACGGCATGACAGTG | | | Ext. | | 68 | 2 min | |  |  |
|  |  |  | | | Final Ext. | | 68 | 10 min | | 1 | |

1. Abbreviations: RACR (Rapid amplification of cDNA ends), GSP (gene specific primer), GRAP (GeneRacer anchor primer), GRNAP (GeneRacer nested anchor primer), Den (denaturation), Ann (annealing), Ext (extension), and Temp (temperature).
