# Supplementary material for: Differential Epigenetic Regulation of TOX Subfamily High Mobility Group Box Genes in Lung and Breast Cancers
Source: PLoS One. 2012 Apr 4;7(4):e34850. doi: 10.1371/journal.pone.0034850 (PMC3319602; doi:10.1371/journal.pone.0034850)
Supplement: Table S3 — Characteristics of TOX high mobility group box family members. (DOC) [file pone.0034850.s004.doc]

**Table S3: Characteristics of TOX high mobility group box family members**

| **Characteristics** | **TOX (TOX1)** | **TOX2** | **TOX3** | **TOX4** |
| --- | --- | --- | --- | --- |
| Chromosomal location | 8q12.1 | 20q13.12 | 16q12.1 | 14q11.2 |
| Number of exons | 9 | 9 | 8 | 9 |
| Coding exons | 9 | 9 | 8 | 9 |
| Genomic size (bp) | 313791 | 154763 | 108890 | 219856 |
| Transcripts | 1 | 4 | 2 | 1 |
| CpG island | yes | yes | yes | yes |
| Size (bp) | 2222 | 2040 | 1209 | 898 |
| Number of CpG | 147 | 172 | 98 | 60 |
| Obs/exp ratio | 0.84 | 0.92 | 0.76 | 0.72 |
| Transcription start site | within | within | within | within |
| Translation start site | within | within | within | within |
