# Supplementary material for: Differential Epigenetic Regulation of TOX Subfamily High Mobility Group Box Genes in Lung and Breast Cancers
Source: PLoS One. 2012 Apr 4;7(4):e34850. doi: 10.1371/journal.pone.0034850 (PMC3319602; doi:10.1371/journal.pone.0034850)
Supplement: Table S4 — Genes with ≥2-fold increase as a result of TOX2 knockdown. (DOC) [file pone.0034850.s005.doc]

**Table S4: Genes with ≥ 2-fold increase as a result of TOX2 knockdown**

| **No.** | **Gene name** | **Fold Change** |
| --- | --- | --- |
| 1 | GDF15 | 6.02 |
| 2 | IFI30 | 3.53 |
| 3 | AK025975 | 3.51 |
| 4 | DHRS9 | 3.45 |
| 5 | CYP1B1 | 3.33 |
| 6 | BEX2 | 3.32 |
| 7 | MT1F | 3.29 |
| 8 | TGFBR2 | 3.27 |
| 9 | WIPI1 | 3.14 |
| 10 | THC2668815 | 3.07 |
| 11 | CPA4 | 3.07 |
| 12 | S100P | 3.03 |
| 13 | IL6R | 2.84 |
| 14 | INSIG1 | 2.83 |
| 15 | F2RL1 | 2.81 |
| 16 | NPC2 | 2.62 |
| 17 | STK40 | 2.59 |
| 18 | IFI6 | 2.55 |
| 19 | ACP5 | 2.49 |
| 20 | UAP1L1 | 2.48 |
| 21 | DNAJB9 | 2.48 |
| 22 | ADAM19 | 2.44 |
| 23 | TP53INP1 | 2.43 |
| 24 | MMD | 2.42 |
| 25 | CYB5R2 | 2.42 |
| 26 | DDAH1 | 2.39 |
| 27 | SLC2A14 | 2.37 |
| 28 | BRI3 | 2.32 |
| 29 | OBFC2A | 2.31 |
| 30 | CR616845 | 2.31 |
| 31 | STEAP1 | 2.29 |
| 32 | SPANXA1 | 2.29 |
| 33 | IFNGR1 | 2.27 |
| 34 | BC010544 | 2.26 |
| 35 | CTSD | 2.26 |
| 36 | DICER1 | 2.25 |
| 37 | AK024926 | 2.24 |
| **No.** | **Gene name** | **Fold Change** |
| 38 | NEU1 | 2.24 |
| 39 | VAT1 | 2.23 |
| 40 | MICB | 2.23 |
| 41 | ENO2 | 2.23 |
| 42 | CTSB | 2.22 |
| 43 | OSTBETA | 2.21 |
| 44 | A_24_P101960 | 2.20 |
| 45 | ITGA5 | 2.20 |
| 46 | ATP9A | 2.19 |
| 47 | C17orf63 | 2.18 |
| 48 | PKIB | 2.17 |
| 49 | BC038556 | 2.17 |
| 50 | TIPARP | 2.17 |
| 51 | STARD4 | 2.16 |
| 52 | AX721087 | 2.16 |
| 53 | SLC16A6 | 2.15 |
| 54 | LIMA1 | 2.14 |
| 55 | ADM | 2.13 |
| 56 | SRPX | 2.13 |
| 57 | CU677606 | 2.13 |
| 58 | ARID3B | 2.12 |
| 59 | AGPAT9 | 2.12 |
| 60 | ANXA10 | 2.11 |
| 61 | HS1BP3 | 2.10 |
| 62 | SPANXD | 2.09 |
| 63 | TFPI | 2.08 |
| 64 | A_23_P147404 | 2.08 |
| 65 | CDKN1A | 2.06 |
| 66 | AGT | 2.05 |
| 67 | A_23_P103951 | 2.04 |
| 68 | HSD17B14 | 2.04 |
| 69 | LGALS3 | 2.03 |
| 70 | EGR1 | 2.02 |
| 71 | GRN | 2.01 |
| 72 | TNC | 2.00 |
| 73 | DHCR7 | 2.00 |
