# Supplementary material for: Differential Epigenetic Regulation of TOX Subfamily High Mobility Group Box Genes in Lung and Breast Cancers
Source: PLoS One. 2012 Apr 4;7(4):e34850. doi: 10.1371/journal.pone.0034850 (PMC3319602; doi:10.1371/journal.pone.0034850)
Supplement: Table S5 — Genes with ≥2-fold decrease as a result of TOX2 knockdown. (DOC) [file pone.0034850.s006.doc]

**Table S5: Genes with ≥ 2-fold decrease as a result of TOX2 knockdown**

| **No.** | **Gene name** | **Fold changes** |
| --- | --- | --- |
| 1 | STARD7 | 0.12 |
| 2 | KIAA0101 | 0.15 |
| 3 | TOX2 | 0.19 |
| 4 | NCEH1 | 0.23 |
| 5 | CHORDC1 | 0.23 |
| 6 | CCL2 | 0.24 |
| 7 | MAD2L1 | 0.29 |
| 8 | ARL2BP | 0.33 |
| 9 | STOM | 0.34 |
| 10 | RRM2 | 0.34 |
| 11 | SPDEF | 0.35 |
| 12 | H2AFX | 0.36 |
| 13 | SUMO3 | 0.36 |
| 14 | KRT4 | 0.36 |
| 15 | C7orf28A | 0.36 |
| 16 | TMEM27 | 0.37 |
| 17 | C7orf28B | 0.37 |
| 18 | DAZAP2 | 0.38 |
| 19 | AK094154 | 0.39 |
| 20 | C6orf62 | 0.39 |
| 21 | MRPS28 | 0.39 |
| 22 | AK026078 | 0.39 |
| 23 | WDR51B | 0.40 |
| 24 | C5 | 0.40 |
| 25 | SLC26A2 | 0.40 |
| 26 | A_24_P170203 | 0.40 |
| 27 | KPNA3 | 0.41 |
| 28 | CCDC80 | 0.41 |
| 29 | PAK2 | 0.41 |
| 30 | GCLM | 0.41 |
| 31 | TRNT1 | 0.41 |
| 32 | DRAM2 | 0.41 |
| 33 | TMEM121 | 0.41 |
| 34 | HIBCH | 0.42 |
| 35 | SPHAR | 0.42 |
| 36 | PACSIN2 | 0.43 |
|  |  |  |
|  |  |  |
|  |  |  |
|  |  |  |
|  |  |  |
|  |  |  |
| **No.** | **Gene name** | **Fold changes** |
| 37 | EI24 | 0.43 |
| 38 | THC2560850 | 0.43 |
| 39 | A_32_P229447 | 0.44 |
| 40 | A_32_P69386 | 0.44 |
| 41 | TMEM206 | 0.45 |
| 42 | PTPN12 | 0.45 |
| 43 | BX107298 | 0.46 |
| 44 | C13orf27 | 0.46 |
| 45 | NHP2 | 0.46 |
| 46 | PREPL | 0.46 |
| 47 | IFFO2 | 0.46 |
| 48 | PHB2 | 0.47 |
| 49 | MDGA1 | 0.47 |
| 50 | MED19 | 0.47 |
| 51 | CHML | 0.47 |
| 52 | URM1 | 0.48 |
| 53 | ACTR6 | 0.48 |
| 54 | SRPK1 | 0.48 |
| 55 | ABCB6 | 0.48 |
| 56 | AV698092 | 0.48 |
| 57 | VASP | 0.48 |
| 58 | RCAN1 | 0.48 |
| 59 | WDR76 | 0.49 |
| 60 | MNS1 | 0.49 |
| 61 | COX16 | 0.49 |
| 62 | TCTEX1D2 | 0.49 |
| 63 | THBS1 | 0.50 |
| 64 | THC2655810 | 0.50 |
| 65 | A_24_P135391 | 0.50 |
| 66 | AI150964 | 0.50 |
| 67 | UBXN2A | 0.50 |
| 68 | RAB8B | 0.50 |
| 69 | TWISTNB | 0.50 |
| 70 | SLC3A1 | 0.50 |
| 71 | KIAA1191 | 0.50 |
