# Supplementary material for: Differential Epigenetic Regulation of TOX Subfamily High Mobility Group Box Genes in Lung and Breast Cancers
Source: PLoS One. 2012 Apr 4;7(4):e34850. doi: 10.1371/journal.pone.0034850 (PMC3319602; doi:10.1371/journal.pone.0034850)
Supplement: Table S6 — Genes with ≥2-fold change as a result of TOX3 knockdown. (DOC) [file pone.0034850.s007.doc]

**Table S6: Genes with ≥ 2-fold change** as a result of TOX3 knockdown

| **No.** | **Gene name** | **Fold Change** |
| --- | --- | --- |
| 1 | HIRA | 3.04 |
| 2 | DISP2 | 2.37 |
| 3 | STMN3 (SCLIP) | 2.30 |
| 4 | DDC | 2.30 |
| 5 | ACTL8 | 2.05 |
| 6 | C6orf176 | 2.02 |
| 7 | C1QTNF6 | 2.01 |
|  |  |  |
|  |  |  |
| **No.** | **Gene name** | **Fold Change** |
| 1 | OSTC | 0.17 |
| 2 | EPHX4 | 0.31 |
| 3 | FGFBP1 (HBP17) | 0.35 |
| 4 | TMEM14A | 0.37 |
| 5 | KCNN4 | 0.39 |
| 6 | CACNG6 | 0.41 |
| 7 | A_23_P66347 | 0.42 |
| 8 | SUSD2 | 0.44 |
| 9 | A_24_P323805 | 0.44 |
| 10 | S100A6 | 0.44 |
| 11 | BECN1 | 0.45 |
| 12 | EFEMP1 | 0.45 |
| 13 | RAB26 | 0.46 |
| 14 | VAV1 | 0.47 |
| 15 | NGEF | 0.47 |
| 16 | LEPROTL1 | 0.47 |
| 17 | LOC440957 | 0.48 |
| 18 | IFITM1 | 0.48 |
| 19 | OASL | 0.48 |
| 20 | ENO3 | 0.48 |
| 21 | VPS25 | 0.49 |
| 22 | CRIP2 | 0.49 |
| 22 | MX1 | 0.49 |
| 24 | AKR1B1 | 0.49 |
| 25 | CKB | 0.50 |
| 26 | C22orf36 | 0.50 |
| 27 | FA2H | 0.50 |
